# Role of Somatostatin Receptor in Pancreatic Neuroendocrine Tumor Development, Diagnosis, and Therapy

**DOI:** 10.3389/fendo.2021.679000

**Published:** 2021-05-19

**Authors:** Yuheng Hu, Zeng Ye, Fei Wang, Yi Qin, Xiaowu Xu, Xianjun Yu, Shunrong Ji

**Affiliations:** ^1^Department of Pancreatic Surgery, Fudan University Shanghai Cancer Center, Shanghai, China; ^2^Department of Oncology, Shanghai Medical College, Fudan University, Shanghai, China; ^3^Shanghai Pancreatic Cancer Institute, Shanghai, China; ^4^Pancreatic Cancer Institute, Fudan University, Shanghai, China

**Keywords:** somatostatin receptor, pancreatic neuroendocrine tumor, somatostatin analog, peptide receptor radionuclide therapy, somatostatin receptor imaging

## Abstract

Pancreatic neuroendocrine tumors (pNETs) are rare and part of the diverse family of neuroendocrine neoplasms (NENs). Somatostatin receptors (SSTRs), which are widely expressed in NENs, are G-protein coupled receptors that can be activated by somatostatins or its synthetic analogs. Therefore, SSTRs have been widely researched as a diagnostic marker and therapeutic target in pNETs. A large number of studies have demonstrated the clinical significance of SSTRs in pNETs. In this review, relevant literature has been appraised to summarize the most recent empirical evidence addressing the clinical significance of SSTRs in pNETs. Overall, these studies have shown that SSTRs have great value in the diagnosis, treatment, and prognostic prediction of pNETs; however, further research is still necessary.

## Introduction

Pancreatic neuroendocrine tumors (pNETs) originate from the neuroendocrine cells in the pancreas and belong to a group of diverse neuroendocrine neoplasms (NENs) (1). Of all the different types of pancreatic neoplasms, pNETs only account for 1 to 2% and are therefore defined as uncommon tumors with a clinical incidence of <1 patient per 100,000 individuals per year (2). Although considered rare, their clinical incidence has been rising from 0.27 to 1.00 per 100,000 individuals in the last 40 years (3). Furthermore, an increasing number of patients are getting diagnosed in earlier stages, possibly due to improved diagnostic methods, in particular endoscopic and imaging techniques (2). Pancreatic NENs (p-NENs) can be classified into two groups according to the presentation of hormone related symptoms: non-functioning (NF-pNENs) or functioning (F-pNENs). A minor fraction (30%) of pNETs are F-pNENs which may release peptides and hormones, for instance vasoactive intestinal peptide (VIP), gastrin, insulin, glucagon, *etc*. (4). Even though most of the pNETs arise sporadically, they have been associated with genetical conditions as well, including tuberous sclerosis, von Hippel Lindau disease, multiple endocrine neoplasia (MEN)-1 (which is also accountable for <5% of insulinomas and 20–30% of gastrinomas), and neurofibromatosis-1. According to their pathological features, pNETs have been categorized as follows: grade 1, which has a well-differentiated morphology and Ki-67 <3%; grade 2, which also has a well-differentiated morphology and Ki-67 3–20%; and grade 3, neuroendocrine carcinomas with Ki-67 >20% and poorly differentiated morphology. The World Health Organization (WHO) introduced the following sub-group to a new grading system for pNETs in 2017: well-differentiated neuroendocrine tumors (NETs) with a Ki-67 >20%, defined as grade 3 pNET, which is clearly different from poorly differentiated neuroendocrine carcinoma, defined as grade 3 pNEC (5, 6). The grade and stage of the pNET determine a patient’s prognosis. Tumors of less than 2 cm usually have a very good prognosis and indicate an indolent grade or biology (7–10). The majority (>80%) of patients with localized tumors, stage I or II, that qualify for resection are cured by undergoing solely surgery. The survival of grade 1 and grade 2 pNETs has significantly improved over the last thirty years, reflected by an increase of around 2 to 5 years in median overall survival (OS) (3). A less promising prognosis is seen in advanced grade 3 pNETs, although it is still superior to poorly differentiated (grade 3) pNECs, with a 5-year survival rate of approximately 29% (11). Surgery is both the main and most significant treatment as well as the only method to cure pNETs. Patients who are unsuitable for surgery can be offered systemic therapy such as peptide receptor radionuclide therapy (PRRT), chemotherapy, targeted therapy, and somatostatin analog (12).

**Figure 1:**
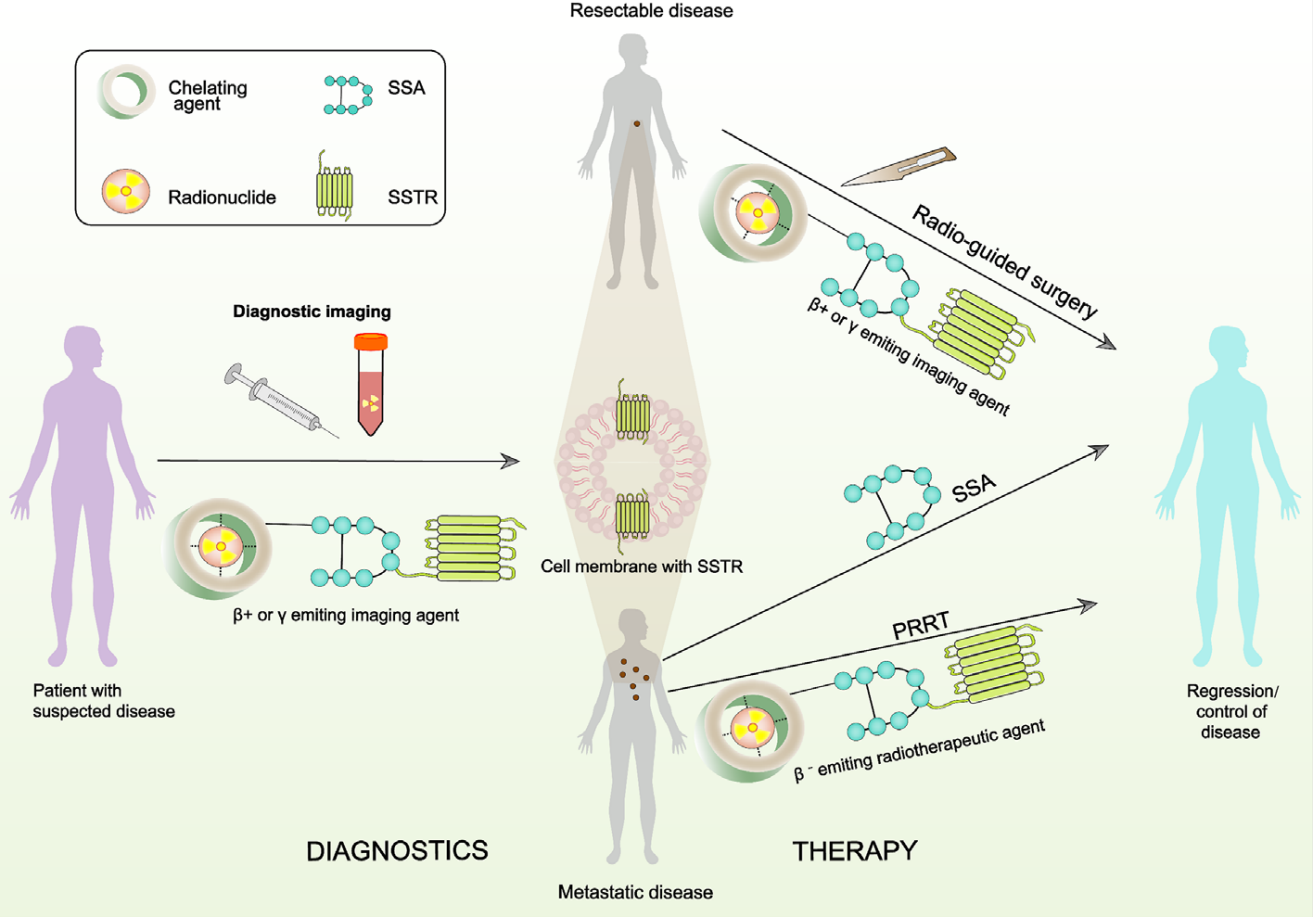
A schematic presentation of theranostics with radiolabeled SSAs that target the SSTRs. The radiopharmaceutical element is comprised of the targeting fraction (SSA) and a chelator that forms a steady composite with the radionuclide. Radiotheranostics consists of diagnostic (panel on the left) and therapeutic (panel on the right) aspects.

Somatostatin receptors (SSTRs) belong to the superfamily of G protein-coupled receptors (GPCRs) and can be activated by their ligands to exert their physiological function (13). Knowledge of SSTRs and their activation has increased over the last 20 years as a result of many clinical and translational studies and has led to the development of novel treatments (14). The clear effectiveness of somatostatin (SST) analogs (SSAs) has been demonstrated in the treatment of numerous diseases including pancreatitis, nephro- or retinopathy as complications of obesity and diabetes, some types of pain, inflammation, and acromegaly (excessive growth hormone produced by the body) (15, 16). In addition, one of the unique features of NETs is the overexpression of SSTRs. Diagnostic and treatment approaches targeting SSTR with SSAs have shown advantages and a promising future prospect (17–20). **Figure 1** represents the theranostic significance of SSTRs in patients with NETs.

In this review, we focused on the diagnostic, prognostic, and therapeutic values of SSTRs in the management of pNETs.

## Biology of SSTRs and SSAs

**Figure 2:**
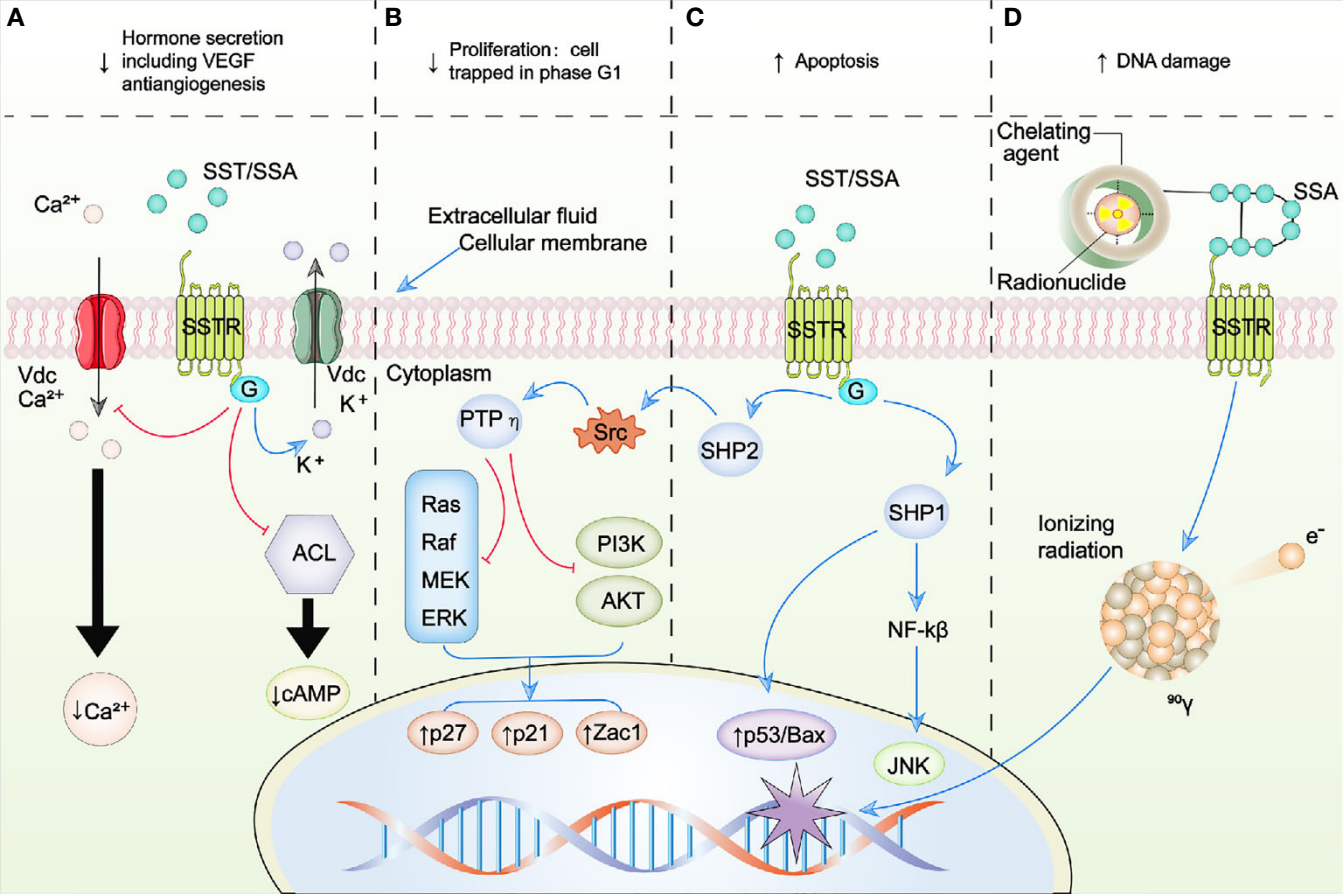
Schematic representation of SSTR-targeted therapy. **(A–C)** represent the intracellular signaling pathways modulated by SSA/SST. **(D)** represents the schematic of peptide receptor radionuclide therapy (PRRT). Blue arrows, activation; red arrows, inhibition; ↑, increase; ↓, decrease; ACL, adenylate cyclase; AKT, protein kinase B; BAX, B-cell lymphoma 2 (BCL2)-associated X protein; Ca2+, calcium; G, G protein; JNK, c-Jun N-terminal kinases; K+, potassium; MEK, mitogen-activated protein kinase kinase; NF*κ*B, nuclear factor kappa B; PI3K, phosphoinositide 3 kinase; PTP*η*, phosphotyrosine phosphatase *η*; raf, rapidly accelerated fibrosarcoma kinase; ras, RAS kinase; SHP1, Src homology phosphatase 1; SHP2, Src homology phosphatase 2; Src, Rous sarcoma oncogene; SSAs, somatostatin analogues; SST, somatostatin; SSTR, somatostatin receptor; Vdc, voltage-dependent channel; VEGF, vascular endothelial growth factor; Zac1, zinc finger protein regulator of apoptosis and cell cycle arrest.

Five subtypes of SSTRs have been discovered (13). Receptor sequences for human SSTRs range in length from 364 amino acids for SSTR5 to 418 amino acids for SSTR3. Unfortunately, crystal structures are not yet available for any SSTR (14). The coding sequences of the genes that encode SSTRs are all intronless, with the exception of SSTR2. The SSTR2 gene could be spliced to generate two distinct receptor proteins, SSTR2A and SSTR2B, which are different in carboxyl termini sequence and length. Only human tissues encompass the unspliced variant of SSTR2A (21). Although SSAs that target SSTR2 and SSTR5 have important therapeutic functions in the treatment of endocrine tumors, it is remarkable that only a few mutations associated with disease have been detected in the somatotropin release-inhibiting factor (SRIF) system, which consist of seven genes (five receptor genes and two peptide precursors). To date, there has been only one report of an acromegaly patient, who is resistant to octreotide treatment and demonstrated a mutation (R240W) of SSTR5 which evidently affected signaling of the receptor (22). SSTR expression can generally be found in tumors and healthy tissues. SSTRs are based in cellular membranes that consist of seven membrane-spanning domains and are connected to the transmembrane potassium ion channels, calcium ion channels, and intracellular enzymes including adenylate cyclase (ACL) and phosphotyrosine phosphatases (PTPs) like phosphotyrosine phosphatase *η* (PTP*η*), Src homology phosphatase 1 (SHP1), and Src homology phosphatase 2 (SHP2). After binding to the SST or SSA, intracellular pathways are activated by SSTRs resulting in antiproliferative and antisecretory effects. In addition, activation of SSTR2 and SSTR3 also exert proapoptotic effects as shown in **Figure 2** (23–26).

Natural SST, also referred to as SRIF, is a cyclic polypeptide of which two isotypes exist (SST-14 and SST-28, which consist of a N-terminal extension). SST functions as an internal regulator of inhibition and is part of the neuropeptide family. SST-14 as well as SST-28 possess a high affinity to bind each of the five related subtypes of SSTRs (14). The hypothalamus can secrete SST, which leads to the inhibition of essential hormones, for instance thyroid-stimulating hormone and growth hormone. Whereas in the gastrointestinal tract, the production of gastric acid is controlled by SST as well as inhibition of the secretion of diverse hormones, namely cholecystokinin, gastrin, glucagon, VIP, secretin, insulin, and gastric inhibitory polypeptide (GIP). In addition, SST can also reduce motility in the gastrointestinal tract and contraction in the gallbladder through the reduction of blood flow and inhibition of exocrine pancreatic secretion (23).

The induction of various biological effects following activation of the SSTR resulted in identifying them as important therapeutic targets. However, the use of native SST as *in vivo* therapy is limited because it has a remarkably short half-life. Thus, many different analogs have been developed that could extend the biological actions of SST, prolong its persistence in the body, and often possess increased efficacy. Among these, the very first octapeptide that was developed was octreotide, which could sustain a half-life of 90–120 min following subcutaneous administration. Subsequently, lanreotide and vapreotide were developed, which were cyclooctapeptide SSAs (27). It has been discovered recently that pasireotide (SOM-230) is one of the very first analogs to demonstrate a strong affinity for the majority of SSTR subtypes, except for SSTR4 (also known as pansomatostatin analog), while octreotide and lanreotide only show a high affinity for SSTR2 and SSTR5 as shown in **Table 1** (29). Consequently, the development of synthetic SSAs promoted the clinical use of radiolabeled SSAs, either in imaging, combined with probes in various clinical practices, or as therapy, with a large number of compounds in clinical research. For instance ^90^Y or ^177^Lu-DOTATATE and ^177^Lu-DOTATOC for PRRT, and SSA labeled with ^68^Ga, such as DOTATOC, DOTATATE, and DOTANOC for somatostatin receptor imaging (SRI) (30). Each of the SSTRs has a high binding affinity to natural SST28 and SST14, while a significant difference is found in the binding affinity of radiolabeled SSAs and synthetic SSAs as shown in **Table 1** (25, 28).

**Table 1:** Somatostatin Analog Affinities.

Somatostatin analog	Affinity (IC_50_ nM)				
	SSTR1	SSTR2	SSTR3	SSTR4	SSTR5
Octreotide	>1000	0.4–2.1	4.4–34.5	>1,000	5.6–32
Lanreotide	>1000	0.5–1.8	43–107	>1,000	0.6–14
Pasireotide	9.3	1	1.5	>100	0.16
In-DTPA-octreotide	>10,000	22 ± 3.6	182 ± 13	>1,000	237 ± 52
Ga-DOTATOC	>10,000	2.5 ± 0.5	613 ± 140	>1,000	73 ± 21
Ga-DOTANOC	>10,000	1.9 ± 0.4	40.0 ± 5.8	260 ± 74	7.2 ± 1.6
Ga-DOTATATE	>10,000	0.20 ± 0.04	>1,000	300 ± 140	377 ± 18
Y-DOTATOC	>10,000	11 ± 1.7	389 ± 135	>10,000	114 ± 29
Y-DOTATATE	>10,000	1.6 ± 0.4	>1,000	523 ± 239	187 ± 50
Lu-DOTATATE	>1,000	2.0 ± 0.8	162 ± 16	>1,000	>1,000

Data from (25, 28).

All data are mean ± SD; IC_50_: half maximum inhibitory concentration (IC_50_ depicts the concentration of a drug needed for in vitro inhibition of 50%; the lower the IC_50_, the stronger the affinity).

## Prognostic Values of SSTR Expression in pNETs

Since SSTRs are present on the surface of tumor cells, it provides a molecular basis for long-acting SSAs to be implemented in therapy and diagnostics; thus, the assessment of SSTR expression in pNETs could be important for diagnostic purposes and SSA-based treatment strategies. In previous research, the expression of SSTR subtypes in pNETs was studied mainly through immunohistochemical methods or reverse transcription PCR (RT-PCR) and only a few by receptor autoradiography (31–40). Although these studies revealed a heterogeneous SSTR expression pattern, it was confirmed in most studies that SSTR2 is the most commonly expressed subtype in pNET (**Table 2**).

**Table 2:** SSTR expression in p-NETs.

Tumor type	SSTR subtype									
	SSTR1		SSTR2		SSTR3		SSTR4		SSTR5	
	mRNA	Protein	mRNA	Protein	mRNA	Protein	mRNA	Protein	mRNA	Protein
p-NET in general	62%(32)	30%(69)	90%(32)	78%(69)	56%(32)	78%(69)	78%(32)	12%(25)	81%(32)	76%(69)
	62%(21)	36%(25)	86%(21)	76%(25)	86%(21)	40%(25)		52%(199)	86%(21)	56%(25)
		53%(199)		55%(199)		29%(199)				34%(199)
Functioning p-NET										
Gastrinoma		30%(33)		100%(33)		79%(33)				76%(33)
Insulinoma		25%(16)		13%(16)		19%(16)		88%(16)		19%(16)
		31%(36)		58%(36)		78%(36)				78%(36)

The data on mRNA expression is obtained from studies that used RT-PCR (31–34). The data on protein expression is obtained from immunohistochemical studies (35–38, 40) that used SSTR subtype-specific antibodies and receptor autoradiography method with subtype-selective SSTR autoradiography (39). The numbers indicate the percentage of tumors that express the corresponding SSTR subtype amongst the total of tumors investigated; the numbers between parentheses represent the total number of tumors included in these studies.

Furthermore, several studies have assessed the potential value of SSTR expression in the prognosis of pNETs. For instance, Okuwaki et al. (41) retrospectively studied 79 pNET patients to evaluate the correlation between outcomes and the intensity of SSTR2a expression (SSTR-2a score from 0 to 3 by immunohistochemistry criteria). The results revealed that the survival rate of patients with a SSTR-2a score of 0 was 58% at 1 year, 51% at 3 years, and 35% at 5 years; patients with a SSTR-2a score of 1 was 88% at 1 year, 74% at 3 years, and 74% at 5 years; patients with a SSTR-2a score of 2 was 94% at 1 year, 80% at 3 years, and 80% at 5 years; and patients with a SSTR-2a score of 3 was 100% at 1 year, 3 years, as well as 5 years. As the results clearly indicate, survival was significantly reduced in patients with a SSTR-2a score of 0 compared to those with a higher SSTR-2a score, implying that assessing the SSTR2 could be valuable in choosing treatment options and estimating future survival. A retrospective study (42) that followed up 116 patients with gastroenteropancreatic neuroendocrine neoplasms (GEP-NENs) showed that the positive expression of SSTR5 and SSTR2 was associated with an improvement in survival. The results indicated that the median OS of patients with a positive expression of SSTR5 and SSTR2 had not been reached yet prior to publication, while the median OS of patients with a negative expression of SSTR5 and SSTR2 was 7.22 and 3.48 years, respectively; however the pNET subgroup was not analyzed exclusively in this study. Another retrospective study, which included 99 pNET patients, demonstrated by univariate analysis that the expression of SSTR2 was correlated to an improvement in OS, with combined survival rates of 97.5% at 1 years, 91.5% at 3 years, and 82.9% at 5 years. In addition, multivariate analysis demonstrated that positive expression of SSTR2 was a greater prognostic indicator of OS than high Ki-67 (43).

Positive expression of SSTR2 (41–44) and SSTR5 (42) has shown a significant correlation with improved OS, indicating its potential value as prognostic marker and imaging, or therapeutic target. However, an agreement on the significance of the expression of SSTR as a prognostic biomarker in pNETs has not been achieved yet and requires additional evaluation in studies of a prospective nature.

## SSTR-Targeted Imaging in pNETs

As discussed above, most well-differentiated pNETs contain and overexpress SSTRs (see **Table 2**) that have a higher binding affinity for these SSAs (SSTR2 > SSTR5 and 3, as shown in **Table 1**). Therefore, Somatostatin receptor imaging (SRI) combined with radiolabeled SSA (^111^In-pentetreotide (Octreoscan)/^68^Ga-DOTA-SSA PET/CT) is increasingly being used as a diagnostic tool when pNET is suspected (45). A review comparing the sensitivity of different imaging methods for pNETs and their metastases in the liver (see **Table 3**) showed that SRI has advantages in sensitivity.

**Table 3:** The sensitivity of different imaging modalities for pNETs and their metastases in the liver.

Imaging modality	Sensitivity (%)				
	Gastrinoma	Insulinoma	pNET <1.5 cm	pNET >2.5 cm	Liver metastasis
CT scan	5–47	20–63	34	50–94	75–100
MRI	10–44	10–85	34	60–95	67–100
US	0–21	26–50	11–33	30–76	15–77
Angiography	15–51	50–60	30–60	60–90	33–86
EUS	40–63	71–94	40–90	82–100	N/A
^111^In-pentetreotide	30–32	33–60	29–30	52–96	90–100
^68^GaDOTATAC PET/CT	68–100	31–90	60–80	68–100	95–100

Data from (46).

pNET, Pancreatic Neuroendocrine Tumor; CT, Computed Tomography; MRI, Magnetic Resonance Imaging; US, Ultrasound; EUS, Endoscopic Ultrasound.

As **Table 3** evidently shows, ^111^In-pentetreotide has a higher sensitivity overall compared to cross-sectional imaging for the two types of primary pNETs (non-insulinomas) as well as a specific advantage in examining the whole body at once and thereby possibly discovering liver as well as distant metastases (47–51). ^111^In-pentetreotide has an overall sensitivity in pNET of 60–80% (52). The use of ^111^In-pentetreotide following cross-sectional imaging led in 39% of patients (with a total range of 16–71%) to an alteration in management (47, 51). Among all the distinct pNETs, SRI is generally not conducted in insulinomas since the sensitivity for ^111^In-pentetreotide in benign insulinomas is considered as low, due to the low levels or absence of SSTR2 and SSTR5 in these type of tumors (53).

Different studies have used a variety of ^68^Ga-labeled SSAs (54, 55). These mainly include ^68^Ga-DOTATATE, ^68^Ga-DOTATOC, and ^68^Ga-DOTANOC (54–57). Although these three possess a different affinity for varying subtypes of SSTRs, they do have a high affinity for SSTR2 in common, and reviews including comparative studies have demonstrated that minor or no obvious differences were observed in their performances (53–55, 58, 59). Multiple published studies, in which the findings of ^68^Ga-DOTA-SSA PET/CT were compared to those of ^111^In-pentetreotide SPECT/CT in the exact same group of patients, concluded that ^68^Ga-DOTA-SSA PET/CT had a significantly higher (which varied from 22 to 46%) sensitivity in the patients ^68^Ga-DOTA-SSA PET/CT 95–100% *vs* SSTR scintigraphy 45–78%) (45, 60–63). It has been recommended most recently to replace SRI with ^111^In-pentetreotide SPECT/CT by ^68^Ga-DOTA-SSA PET/CT since it has a higher diagnostic accuracy and sensitivity and requires a smaller dose of radiation (45, 54, 55). However, a recently published meta-analysis, which only included pNET patients, that evaluated the detection of the primary lesion and its primary staging with ^68^Ga-DOTA-SSA PET/CT demonstrated that the pooled specificity and sensitivity for identifying primary pNET was 95 and 79.6%, respectively (64). This sensitivity was lower compared to the results of other meta-analysis/series, which included patients with different type of NETs, and demonstrated a mean sensitivity of 92% (range between 68 and 100%), a relatively high mean specificity of 88% (range between 50 and 100%), and a high mean accuracy of 93% (range between 90 and 97%) (50, 54, 55, 65–69). These differences might be correlated to the PET/CT’s spatial resolution that can cause restriction in the identification of minor pancreatic lesions and the inclusion of higher histopathological grades of pNETs in these studies which could have resulted in an increase of false-negative outcomes due to a lower expression of SSTR (70). Moreover, the inclusion of insulinoma patients could also have contributed to these differences due to their restricted expression of SSTR in comparison with carcinoids, the most common histopathological subtype of GEP-NET, resulting in the potential reduction of SSTR-PET sensitivity (71).

^68^Ga-DOTA-SSA PET/CT also has a high sensitivity for identifying metastases in liver, lymph nodes, and distant ones (bone, *etc*.), which has a great influence on treatment, prognosis, and OS (50, 68, 72–75). Various studies (56, 76–79) have demonstrated that the tumor standardized uptake value (SUV) of ^68^Ga-DOTA-SSA PET/CT is related to progression-free survival (PFS), Ki-67, tumor progression, and tumor grade/differentiation. Another study also found that the SUV of ^68^Ga-DOTA-SSA PET/CT in NET patients is related to the expression of SSTR2 and can serve as a distinct predictor of OS (44). In addition, it has been demonstrated that the SUV of ^68^Ga-DOTA-SSA PET/CT correlates with the uptake amount of radioligand in PRRT (80), and a maximum cut-off of 16.4 could predict responding lesions with a specificity of 60% and sensitivity of 95% (81).

SRI has also demonstrated its value in radioguided surgery (RGS). RGS makes use of radiopharmaceuticals that are uptaken by tumor tissues by preference. Studies found that RGS combined with ^68^Ga-DOTATATE in GEP-NET patients showed feasibilities in guiding the removal of lymph node metastasis and both intraoperative evaluation as well as establishing the correctness of surgical margins. In addition, it could also be valuable in the identification and removal of minor tumors that were invisible or not palpable in recurrent NET patients, in whom the surgical area is covered with scar tissue (82, 83).

These studies suggest that SRI with radiolabeled SSAs have an essential role in identifying the primary tumor, initial staging, restaging, prognosis, intraoperation guidance, and evaluation of the response to treatment in pNET patients. Moreover, SRI can differentiate whether or not patients are suitable for treatment with PRRT. This is a key feature of targeting SSTRs because it provides the opportunity to personalize treatment (also referred to as theranostic approach as shown in **Figure 1**).

## SSTR-Targeted Therapy in pNET

The therapeutic value of PRRT and SSAs in NETs relies on the biological foundation of SSTR expression on the NET’s surface (see **Figure 2**).

### SSA in the Treatment of pNET

#### Antiproliferative Effects

SSAs function through targeting SSTRs (84). The most studied SSAs are lanreotide autogel and octreotide long-acting release (LAR), which primarily target SSTR5 and SSTR2. Whereas the newest SSA, pasireotide, can target a broader scope of SSTRs, including SSTR1, 2, 3, and 5 as shown in **Table 1** (85, 86). Due to their anti-secretory effects, SSAs were previously only used to regulate symptoms (84). However, at present their anti-proliferative effect has been widely confirmed (87).

The PROMID clinical trial was the first to provide solid evidence of the anti-proliferative effect (88, 89). This study was a double-blind, placebo-controlled, prospective phase III randomized controlled trial (RCT), in which the effect of octreotide LAR was evaluated in patients who had a metastatic or locally advanced, non-treated grade 1 midgut NET, or an idiopathic NET. The results showed that the increase in median time to progression (TTP) of the tumor was clinically and statistically significant (placebo 6 months *vs.* octreotide LAR 14.3 months and hazard ratio (HR) of 0.34 (95%-CI 0.20–0.59; *p* = 0.000072). The patients in this study who were in the placebo group were permitted to go over to the octreotide LAR group if progression occurred, which is probably the primary cause of TTP differences not resulting in an improvement of the OS. Even though no pNET patients were included in this RCT, the results were still regarded as powerful and led to the addition of octreotide as treatment in pNET patients to the ENETS guidelines (19, 90). This was further confirmed by a few small phase II studies and retrospective series that demonstrated the anti-proliferative effect of octreotide LAR in patients with a pNET, of which a majority were low Ki-67 NETs (as longer lasting responses were observed in patients with a low Ki-67 of less than 10) (91). The CLARINET study was a crucial phase III trial, in which the effects of SSA in pNET patients was evaluated (20, 92–94). This randomized, placebo-controlled, and double-blinded study assessed lanreotide autogel in patients who had metastatic or locally advanced, well-differentiated, and non-functioning (except for gastrinomas) GEP-NETs with a low Ki-67 of less than 10%. The (core) study duration was 96 weeks, which was followed by an open label extension (OLE) component. The majority of the included patients were treatment-naïve (84% in both groups) and were in a steady disease state during baseline (95 and 96% in the placebo and lanreotide group, respectively). The findings demonstrated an advantage in regard to PFS with a HR of 0.58 (95%-CI 0.32–1.04 in the core study) (92) and median PFS of 29.7 months in the pNET group (core study and OLE as a whole). The advantage in PFS was seen irrespective to tumor burden (20). Despite the poor response rate (2%), stabilization of disease was still high (64%), which resulted in a great disease control rate (DCR) of 66%. Data on the patients, during OLE, that crossed over to the lanreotide autogel group due to disease progression under placebo and were initially already in that group without disease progression at week 96 (n = 88) showed that, interestingly, 50% of these patients had pNETs (93). The median PFS of pNET patients was 29.7 months, which was shorter compared to the median PFS of all the included patients (38.5 months) (20). A large number of studies have tried to enhance the anti-tumor ability of SSAs by developing novel SSAs like pasireotide LAR (95) or compounds of SSAs combined with other anti-tumor media like everolimus, as demonstrated in the COOPERATE-1 study (96). However, these studies have not yielded any successful results and the clinical use of SSAs in the treatment of pNETs is, at present, still restricted to single agent approaches.

#### Anti-Secretory Effects

In patients with malignant insulinoma, SSAs are mainly used as second-line medical therapy to regulate hypoglycemia. A previous study has demonstrated that octreotide can be successful in regulating hypoglycemia in a majority of insulinoma patients (97). In addition, pasireotide could be considered as an alternative treatment choice in malignant insulinomas and subsequent recurrence of hypoglycemic incidents since it is capable of regulating hypoglycemia in insulinomas that are resistant to other therapies, such as octreotide LAR, everolimus, and chemotherapy (98). However, SSAs can also exacerbate hypoglycemia through the inhibition of counter-regulatory processes, such as GH and glucagon, in insulinomas that do not express SSTRs (99). High dosages of proton pump inhibitors can effectively decrease oversecretion of gastric acid, although it cannot decrease the abnormal increase of enterochromaffin-like (ECL) cells. On the contrary, multiple studies have shown that the use of SSAs, such as lanreotide and octreotide LAR, in type 1 gastric NETs (related to chronic atrophic gastritis) and type 2 (related to the Zollinger–Ellison syndrome) can suppress the secretion of gastrin and decrease the tumor burden. Their results show that in 50–100% of gastrinomas, gastric secretion is either decreased or normalized, which resulted in the stabilization of the tumor in 47–75% of included patients. Furthermore, SSAs may be capable of inhibiting hyperplasia of ECL cells or the growth of type 2 gastric NETs (100–102). Lanreotide and octreotide have demonstrated the ability to quickly reduce diarrhea and migratory necrolytic erythema in glucagonoma patients, despite the sustained rise of glucagon levels in the serum (103–105), whereas pasireotide has been proposed as a suitable treatment approach in first-generation glucagonomas resistant to SSAs. Treatment with octreotide, as an adjuvant, in the rare vipomas was successful in decreasing VIP levels in serum and regulating diarrhea (106–108). Even though it seems contradictory to use SSAs in the treatment of somatostatinomas, a study has shown that octreotide relieved the associated symptoms and successfully decreased the levels of SST in the plasma of three patients (109).

### PRRT in the Treatment of pNETs

The effectiveness of PRRT in NETs is based on the biologic foundation of SSTR expression on the NET’s surface. PRRT is comprised of a radionuclide (*e.g.*, *β*-emitters Lutetium-177 [^177^Lu] and Yttrium-90 [^90^Y], *α*-emitter Actinum-225 [^225^Ac]) which is connected to a chelator (DOTA) that is bound to a SSTR ligand, for instance [Tyr3] octreotide or [Tyr3] octreotate (110). This composite is intravenously given after which the ligand, [Tyr3] octreotate, first connects to the cell surface’s SSTR and then supplies emission of *β*^−^ radiation with a span of 12 mm for ^90^Y and 2 mm for ^177^Lu (111). Among the compounds that have been studied, β-emitters, ^90^Y-DOTATOC and ^177^Lu-DOTATATE, have been the most widely used clinically. However, recently several clinical studies using PRRT with *α*-emitters have demonstrated its strengths compared with *β*-emitters, which will be discussed below in more details.

#### Anti-Tumoral Efficacy

It is worth noting that no prospective and randomized phase III trials have been conducted with PRRT in pNETs. Although, the NETTER-1 trial is the biggest study to date that evaluated the effects of PRRT, it unfortunately did not include any pNET patients (18). However, several non-randomized studies have been reviewed and they provided retrospective as well as prospective data on evaluation the use of PRRT with ^177^Lu-DOTATATE in pNET patients (112, 113). The results showed a median objective response rate (ORR) of 58% (with a range between 13 and 73%), a median DCR of 83% (with a range between 50 and 94%), a median OS between 42 and 71 months, and a median PFS ranging between 25 and 34 months. A retrospectively conducted study including 74 GEP NET patients demonstrated that a more elevated ORR (adjusted SWOG criteria) of 73 *vs* 39% (*p* = 0.005) was found in pNET patients. This group of patients also seemed to have a longer median OS (57 *vs.* 45 months); however, this finding was only observed in the univariate analysis (*p* = 0.037) and not in the multivariate analysis (*p* = 0.173) (112). Another retrospective study that included 310 GEP-NET patients showed that the patients with functional pNETs had a decreased disease-specific survival in comparison to patients with non-functional GEP-NETs (33 *vs.* >48 months, respectively, *p* = 0.04) (114). These findings were further underwritten by the outcomes of another retrospective study which had 68 patients included. The results demonstrated a poorer median OS in functional pNETs compared to non-functional pNETs with univariate analysis (45 *vs.* 63 months, respectively, *p* = 0.045); however, these findings did not show statistical significance in the multivariate analysis (*p* = 0.506) (115).

To date, the largest study that evaluated ^90^Y-DOTATOC has been a prospective phase II trial in which 342 pNET patients were enrolled (divided in functional pNET, n = 47 and non-functional pNET, n = 295). Nearly 50% of the pNET patients (ORR = 47%, according to the RECIST criteria) had tumor response. In addition, the study revealed a mean OS of 60 months in the group of nonfunctional pNET patients (116).

Although PRRT with *β*-emitters has shown a good clinical effect, recently, a more promising radionuclide, *α*-emitters has attracted increased attention in radionuclide therapy (117, 118). Radioisotopes that emit *α*-particles which have higher energy and shorter penetration range in comparison with *β*-particles, induce a higher probability of double strand breaks and minimum damage to surrounding healthy tissue (119, 120). These *α*-emitters have demonstrated promising therapeutic effects in a few pre-clinical *in vitro* (121–123) or *in vivo* (124, 125) studies. Currently, the only clinical experience with ^213^Bi-DOTATOC included seven patients with advanced NETs with liver metastases who were refractory to treatment with ^90^Y-DOTATOC or ^177^Lu-DOTATOC (117). It demonstrated lower toxicity, better specific tumor binding than with *β*-irradiation, and partial remission of metastases. Two years after receiving ^213^Bi-DOTATOC targeted alpha therapy (TAT), all seven patients were still alive. A study with another type of *α*-emitters, ^225^Ac, had included 10 patients with progressive NETs after *β*-PRRT. In line with ^213^Bi, ^225^Ac-DOTATOC was well tolerated and effective (126). Another recent study with ^225^Ac-DOTATATE confirmed the potential of these radiotracers as an additional, and valuable, treatment option for patients who are refractory to ^177^Lu-DOTATATE therapy. The included 32 patients, who previously received ^177^Lu-DOTATATE therapy, were treated with ^225^Ac-DOTATATE. Of them, 24 patients were assessed as responsive, with nine as stabilized disease and 15 partial remissions (127). The clinical experience with TAT in NETs has shown very promising results even in patients refractory to treatment with *β*-particles. However, further investigations are needed due to the limited amount of clinical evidence.

#### Efficacy in Hormone-Related Symptoms

There have been two studies that studied PRRT as treatment of gastrinomas (128, 129). In one of these studies 11 gastrinoma patients were assessed, and the results indicated that every patient experienced improvement of their symptoms although, the median OS was just 14 months (129). In contrast, the findings of the other study,which assessed 36 gastrinoma patients, revealed an ORR of 30% as well as a clinically observed response rate of 16% (128). In addition, the median OS was reported to be 45 months in the patients that were considered as responders. In terms of malignant insulinomas, there is a limited amount of data by means of case reports or series that indicate a positive result of PRRT in stabilization of disease as well as hypoglycemia (130, 131). Another recently published retrospective study, which had 34 functional pNET patients with metastasis and persistent hormonal symptoms included in it, reported that most patients (71%) showed a significant improvement in terms of the functional syndrome and 80% of them showed a decrease in the circulating levels of related hormones. Following PRRT, the outcomes demonstrated a median PFS of 18.1 months, which was correlated to a coexisting improvement of quality of life (132).

Overall, PRRT can be considered as a real innovation in the treatment of NETs. Even though randomized and prospective data of PRRT in pNETs is limited, the data that is available today indicates that it is an effective treatment for pNETs and should be studied further.

## Future Prospective

### SSTR Antagonists in Imaging and Therapy

SRI and PRRT use radiolabeled SSAs (see **Table 1**), which are only SSTR agonists as mentioned previously, mainly because it is believed in general that agonists would be the most suitable for imaging since they are internalized, while SSTR antagonists are not (133). It has been uncovered recently that SSTR antagonists with radiolabeling produce more superior imaging than SSTR agonists with radiolabeling (133, 134). A study conducted *in vitro* with SSTR3 antagonists revealed that it detected 76-fold more sites of binding in comparison to the SSTR3 agonist (134). Thereafter, a few studies which only included a minor amount of NET patients (pNETs as well as GI-NETs were included) showed that SSTR2 antagonists with radiolabeling, *i.e.*, ^111^In-DOTA-BASS and ^68^Ga-OPS202 (^68^Ga-NODAGA-JR11), demonstrated more superior imaging of the tumor and higher sensitivity than SSTR2 agonists with radiolabeling (28, 133–136). These results have led to the option of using ^177^Lu-radiolabeled SSTR2 antagonists in PRRT instead of ^177^Lu-radiolabeled SSTR2 agonists. The results of another preclinical study (137) conducted *in vivo* with SSTR2 positive cells and in mice with tumors, showed that the tumor uptake was five times more with SSTR2 radiolabeled antagonists, ^177^Lu-DOTA-JR11, compared to the SSTR2 radiolabeled agonist, ^177^Lu-DOTA-octreotate, which led to a longer delay in growth. When research using these two SSTR2 radiolabeled compounds were expanded to four advanced NET patients (134), the ^177^Lu-DOTA-JR11 provided 1.7–10.6-fold higher tumor uptake dose compared to the agonist, ^177^Lu-DOTA-octreotate, which resulted in a partial remission in half of the enrolled patients. These findings indicate that SSTR2 radiolabeled antagonists have the potential of being an improved agent in comparison to SSTR2 radiolabeled agonists in pNET/NET imaging and PRRT.

## Conclusion

Although pNET is a highly heterogeneous disease, SSTR is expressed in most pNETs, which provides the opportunity for promising approaches and strategies in diagnosing, treating, and predicting the prognosis of pNET patients. In the previous few decades magnificent progress has been made in the clinical significance of SSTRs in pNETs. SRI and therapies with radiolabeled SSA have shown significant value in clinical practice and has been recommended in various guidelines. However, an even more promising agent, namely radiolabeled somatostatin antagonists, has shown its superiority compared with agonists. Despite the accumulation of evidence that SSTR- targeted or related therapies (*e.g.*, SSAs and SSTR-targeted PRRT) are safe and effective options for refractory or unresectable pNETs, most SSTR-targeted therapies target SSTR2, and for those SSTR2-negative patients, more effective therapeutic approaches targeting other SSTRs are urgently needed. More and larger randomized prospective trials, conducted in multiple centers with a long-term follow-up are desperately needed as well. In addition, research deciphering crystal structures for the five SSTRs are also needed, in particular to uncover the exact signaling pathways of SSTR ligands and SSAs that underlie its antitumor effects and to facilitate the development of novel SSTR subtype-selective agents, along with the detection and selection of appropriate candidate patients who could benefit from these therapies.

## Author Contributions

SJ conceived of the presented idea. YH and ZY wrote the manuscript. FW and YQ searched the literature. XX and XY supervised the project. All authors contributed to the article and approved the submitted version.

## Funding

This work was supported by grants from National Science Foundation of China (No.81871950 and 81972250); Scientific Innovation Project of Shanghai Education Committee (2019-01-07-00-07-E00057); and National Science Foundation for Distinguished Young Scholars of China [81625016], Shanghai Municipal Commission of Health and Family Planning (No. 2018YQ06).

## Conflict of Interest

The authors declare that the research was conducted in the absence of any commercial or financial relationships that could be construed as a potential conflict of interest.
